# Bioengineering Approaches and Novel Biomaterials to Enhance Sternal Wound Healing after Cardiac Surgery: A Crosstalk between Innovation and Surgical Practice

**DOI:** 10.3390/jfb15090254

**Published:** 2024-08-31

**Authors:** Chiara Ferrisi, Francesco Loreni, Antonio Nenna, Omar Giacinto, Mario Lusini, Massimo Chello

**Affiliations:** Unit of Cardiac Surgery, Fondazione Policlinico Universitario Campus Bio-Medico, 00128 Rome, Italy; chiara.ferrisi@unicampus.it (C.F.); francesco.loreni@unicampus.it (F.L.); o.giacinto@policlinicocampus.it (O.G.);

**Keywords:** cardiac surgery, mediastinitis, sternal, sternotomy

## Abstract

Median sternotomy and steel wires for sternal closure are the standard approach for cardiac surgery. An incomplete repair associated with chest wall motion, especially in the presence of predisposing factors, can lead to life-threatening deep sternal wound infection, also known as mediastinitis, in 2–5% of cases. Despite current antibiotic and surgical treatments, mediastinitis is associated with a 10–40% mortality rate and a significant increase in morbidity and hospital stay. High mortality and difficult treatment appear to be due to bacterial biofilm, a self-produced extracellular polymeric product that incorporates host tissue and is responsible for the failure of immune defenses and standard antimicrobial therapies. Nanostructures are an effective strategy to enhance the healing process, as they establish a favorable environment for the neosynthesis of the extracellular matrix, supporting tissue development. Synthetic polymers have been proven to exhibit suitable biodegradable and mechanical properties, and their biofunctionalization to enhance cell attachment and interaction with the extracellular matrix is being widely investigated. The use of antibiotic treatments suspended in poly-D,L-lactide and polyethylene oxide and electrospun into nanofibers, or in sponges, has been shown to inhibit bacterial biofilm production. Additionally, growth factors can be incorporated into 3D bioresorbable scaffolds with the aim of constituting a structural and biological framework to organize and expedite the healing process. Therefore, these combined approaches may change the treatment of mediastinitis in the near future.

## 1. Introduction

Median sternotomy is the most frequent approach to cardiac surgery, and multiple factors play a crucial role in the outcome of the procedure. Patients’ clinical characteristics are crucial elements in order to avoid post-sternotomy complications; however, the experience of surgeons plays a key role. Post-sternotomy complications include sternal separation, mediastinitis, and subcutaneous infection, which can lead to longer hospital stays. Surgeons perform several sternal closure techniques to avoid complications and to guarantee a safe healing process, since pioneering techniques with wires at the beginning of the XX century. 

Currently, stainless steel wires are the primary method used for sternal closure thanks to their low costs and training requirements. Different wiring closure techniques can be performed for sternal closure, such as single wires that are trans-sternal, peri-sternal, or alternating; figure-of-eight; modified figure-of-eight; or longitudinal parasternal (Table 1) [1].

Overall, the use of innovative sternal closure methods and alternative materials for hemostasis represent significant advancements in cardiac surgery, particularly for patients at high risk of postoperative complications. Innovative techniques and materials have been developed to improve sternal closure and provide bone healing. 

This review highlights new advancements in sternal closure methods, hemostatic agents alternative to bone wax, bone cement, growth factor therapy, and tissue engineering, showing positive results in promoting sternal stability, reducing complications, and enhancing the overall healing process.

## 2. Patients and Risk Factors for Postoperative Complications

The wound infection could be classified into two different types: (i) superficial, involving skin and subcutaneous tissues; and (ii) deep infection or mediastinitis, when an infection reaches the mediastinum.

Typical microbial etiology is bacterial [2,3]. Mainly, Staphylococcus aureus and Coagulase-negative Staphylococcus represent the main agents’ source of infection. However, Gram-negative bacteria may also be responsible for infection, especially Escherichia coli, Pseudomonas aeruginosa, and Klebsiella, while fungal infections are rare. When mediastinitis occurs, prolonged hospitalization, higher mortality rates, and increased cost of care have been shown [3]. 

In order to avoid deep infections and systemic sepsis status, it is important to recognize early signs of inflammation, such as fever, tenderness, elevation of leukocytes, and acute phase reactants. As Balachandran’s review showed, the mortality rate reaches 47% when sternal complications are undetected, even if the incidence of these complications is relatively low, ranging from 0.4% to 8% [4].

It is important to identify patients at high risk of sternal complications to recognize the most appropriate sternal closure as well as to manage the postoperative period, including mobilization, prescription of post-thorax vests, and rehabilitation. Moreover, a correlation between sternal instability and infection has been proven [5]. 

**Figure 1:**
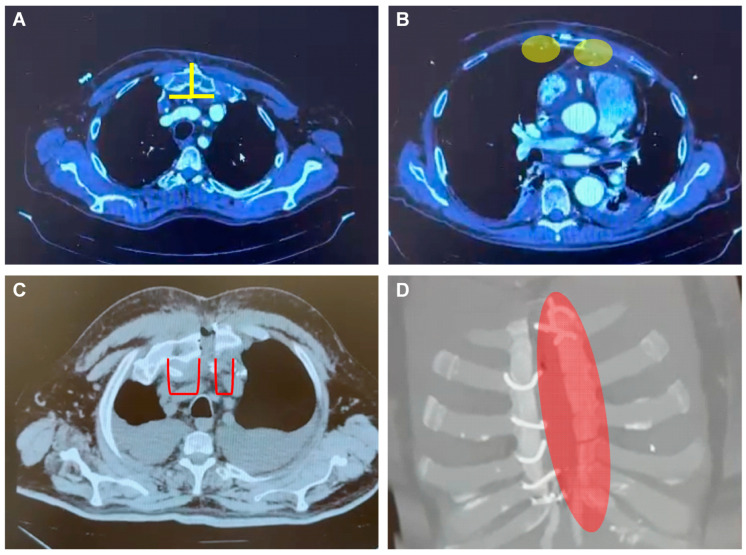
Crucial risk factors for postoperative complications. Normal anatomy and pathophysiology after cardiac surgery: median sternotomy ((**A**), yellow lines) and position of the mammary artery ((**B**), shaded in yellow). Crucial risk factors for postoperative complications include paramedian sternotomy ((**C**), red lines) in which the two parts of the sternum are not symmetric with tension imbalance resulting in impaired healing and respiratory distress, besides pleural effusion that is frequently observed. Also, sternal fractures ((**D**), shaded in red) especially after paramedian sternotomy or mammary artery harvesting are difficult to treat with conventional closure systems and increase the risk of mediastinitis.

The main risk factors for sternal infection may be divided into preoperative, perioperative, and postoperative (Figure 1, Table 2) [4,5]. The preoperative risk factors are the following: female gender; diabetes mellitus; obesity; severe chronic obstructive pulmonary disease; active smoking; osteoporosis; and corticosteroid use. The perioperative factors include paramedian sternotomy; sterility breaks; prolonged operative time; poor closure technique; and bilateral internal mammary harvesting. The postoperative risk factors are blood transfusion; reoperation; and longer hospital stay. 

### 2.1. Preoperative Risk Factors

Copeland and colleagues [6] found a positive relationship between macromastia and a higher incidence of sternal infection. In fact, women with larger breasts are affected by an inferno-lateral tension across their sternotomy, leading to sternal dehiscence, when it is unsupported. 

However, even male patients are predisposed to developing sternal infection, especially those with a large chest circumference or obese patients. The elevated risk of infection, actually, may be linked to the lower vascularity of fatty tissue, resulting in compromised wound nutrition. 

Smoking has been recognized as an independent risk factor for sternal infection [7,8]. Nicotine should affect the healing process by harming smaller blood vessels, decreasing collagen production, and altering the remodeling of connective tissue. Furthermore, prolonged elevated blood glucose levels may lead to a higher rate of infections, because of a dysfunction of both innate and adaptive immune systems. Because of this, diabetes patients are more likely to develop infections due to insufficient responses to external attacks. Mainly, Trick and his colleagues [9] discovered a deep relationship between diabetes mellitus and mediastinitis related to methicillin-resistant Staphylococcus aureus. 

### 2.2. Intraoperative Risk Factors

The internal mammary artery (IMA) supplies the anterior chest wall with its branches. The type of IMA graft used has been linked to a greater risk of sternal dehiscence, especially for high-risk patients. Harvesting these arteries may result in sternal hypoperfusion, compromising tissue healing. De Paulis and his colleagues [10] recommended that the type of IMA graft used could influence the incidence of sternal complications. Furthermore, the use of pedicled bilateral IMA may increase the risk of infection compared with a skeletonized graft.

### 2.3. Postoperative Risk Factors

Reoperation for postoperative complications may prolong the time of exposure of the mediastinum to airborne pathogens, leading to deep sternal wound infections [3]. 

Patients who underwent cardiac surgery may require a blood transfusion, contributing to host immunosuppression, as several studies showed, although this depends on the number of blood replacements performed. Hence, patients with several blood transfusions are more likely to develop deep or superficial wound infections.

## 3. “Traditional” Alternatives to “Traditional” Wires: Current Evidence

**Figure 2:**
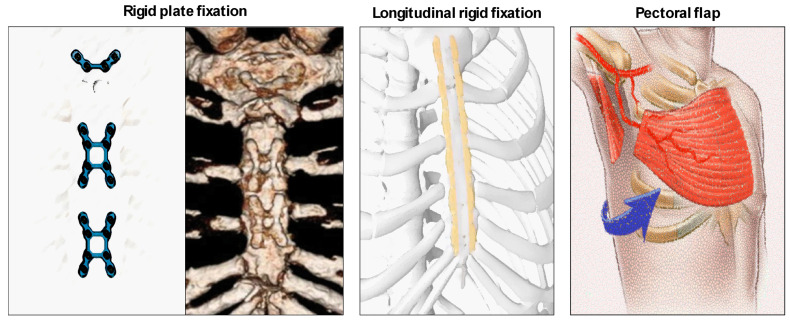
“Traditional” alternatives to “traditional” wires: rigid plate fixation (**left**), longitudinal fixation (**center**), and pectoralis muscle flap (**right**).

As an alternative to wires, sternal bands or plates and polymer cable ties should be used in order to prevent sternal complications mainly in high-risk patients (Table 1, Figure 2).

A randomized trial by Allen [11] showed how the use of rigid plate fixation (RPF) led to better outcomes in terms of sternal wound complications, deep and superficial wound infections, and sternal healing than wire cerclage (WC) after 6 months from the surgery. A lower rate of post-cardiac surgery complications leads to clinical and economic benefits for patients as well as for hospital costs, reducing rehospitalization. As Allen reported, the average cost of rehospitalization was USD 45,532; hence, it is mandatory to perform a sternal closure “tailor-made” for all patients. Although the initial hospital costs rise when RPF is used compared with WC, these costs are more than recovered after 6 months. Rigid plate fixation should be considered as a valid alternative to sternal closure, leading to better outcomes without increasing costs [12].

The same results were discussed by Madjarov [13], comparing wire cerclage and longitudinal rigid sternal fixation techniques in high-risk patients. The longitudinal plates, spreading an equal pressure of the wires alongside the lateral edge of the sternum, guarantee great stability of the sternum in obese or immunosuppressed patients with osteoporosis. Moreover, an additional aspect analyzed was the reduction in postoperative chest pain when RPF was performed compared with WC. RPF, as Madjarov underlined, is also a solution for the treatment of dehiscence, instead of using a pectoralis muscle flap or mesh graft. When muscular flaps were performed, the two halves of the sternum remained separated, carrying on chronic postoperative pain. The integrity of the sternum (bone stabilization), instead, had a strong impact on the mechanics of breathing and chest pain, showing positive short- and long-term outcomes [13].

However, the management of the sternal wound is really challenging for surgeons. Several surgical techniques have been proposed with different outcomes, but the strategy depends on the surgical team’s experience and the clinical characteristics of the patient [5].

In case of complications arising from delayed wound healing or infection, surgical procedures for reconstruction of sternal dehiscence include debridement and muscle flaps. Two different surgical strategies should be performed: single-stage procedures, when the debridement is followed by muscle flap closure, and two-stage procedures, when a vacuum-assisted closure (VAC) is placed before reconstruction.

VAC therapy enhances wound healing by increasing sternal blood flow, avoiding bacterial loads, improving granulation tissue formation, and stabilizing the sternum [14,15].

The muscle flap closure can use major muscles, like the omentum, or a combination of the muscle and fat. The omental flap is not commonly used because it requires laparoscopy for its harvesting, and many complications, such as abdominal or diaphragmatic hernia, frequently occur. Several muscles should be used for reconstruction, but the pectoralis major muscle (PM) and rectus abdominis muscle (RAM) are mainly preferred. The use of the PM guarantees better stabilization of the thoracic wall than wire cerclage. Moreover, reconstruction with the bilateral pectoralis major muscle, combined with the anterior layer of the RAM fascio-cutaneous sheath, showed a greater long-term outcome than using only the PM. Postoperative complications (such as hematoma, total or partial flap loss, chronic chest pain, arm or shoulder weakness, and re-exploration for wound necrosis) could lead to a prolonged hospitalization time and a high mortality rate [16].

### Mechanical Properties

The mechanical properties of osteosynthesis materials and methods are crucial in cardiothoracic surgical procedures, where the integrity of the sternum is vital for patient recovery. The sternum, being subjected to various mechanical forces during movement and respiration, can experience tension, leading to sternal dehiscence. According to recent studies, the improper distribution of forces during the closure of the sternum can significantly increase the risk of this complication. The closure techniques should be carefully selected to ensure they can withstand the tensile forces exerted on the sternum. Obviously, the tension forces acting on the sternum are influenced by several factors such as body mass index (BMI), or postoperative movements. Several studies involving cadaveric experiments examined the various forces (longitudinal, lateral, or torsional) that exert significant stress on the sternum, as well as the region of the sternum most prone to instability [17]. Data revealed the following: (i) forces applied laterally resulted in greater sternal motion compared to other angles, and (ii) the lower region of the sternum, known as the xiphoid region, is more susceptible to dehiscence than the manubrium [17].

As discussed above, there are different sternal closure techniques, such as wire cerclage, plating, and rigid fixation, offering various advantages and risks based on their mechanical properties [18]. For instance, the use of titanium plates has been noted for their strength and ability to distribute stress evenly across the sternal edges, minimizing localized tension. However, their rigidity might also lead to complications related to rigid fixation, limiting physiological mobility, and potentially increasing the risk of pseudoarthrosis. An in vitro simulation study compared the titanium cable system and stainless steel wire, showing that the first one provides a higher resistance when subjected to different tensile forces, guaranteeing better sternal fixation [18]. 

Moreover, similar studies were conducted on 3D-printed bone models of a sternum of dogs, highlighting the superiority of stainless steel bone staples over polydioxanone sutures and nitinol bone staples. The bone staples, which have undergone mechanical forces, show minimal displacement of the two halves of the sternum [19]. 

The choice of screws and plates, instead, influences and reduces load distribution and stress concentrations around the osteosynthesis site, preventing potential failures, especially when the cortical shell is thin [20]. Alternatively, wire cerclage offers a more flexible approach that may better accommodate the biomechanical stresses experienced by the sternum. Yet, wire techniques could predispose sutures to failure, heightening the risk of dehiscence if not executed with precise tension. 

Several aspects should be considered when osteosynthesis is performed. It is important to focus on materials that provide the optimal balance of strength and flexibility, and the characteristics of the patients, in order to minimize the risk of complications post-surgery. 

## 4. New Methods and Devices for Sternal Closure

### 4.1. Fixsorb Wave

**Figure 3:**
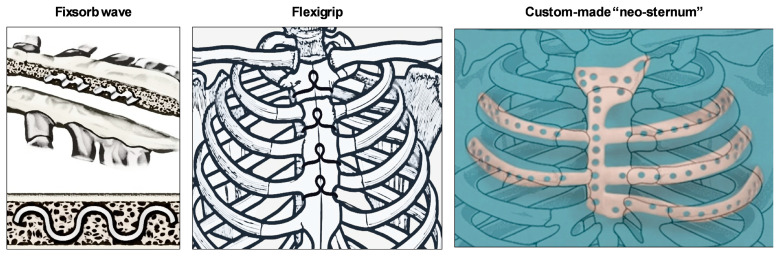
New methods and devices for sternal closure: Fixsorb wave (**left**), Flexigrip (**center**), and custom-made neo-sternum (**right**).

A randomized controlled study by Oishi highlighted the efficacy of a new sternal fixation device called “Fixsorb Wave” (Figure 3), a corrugated plate made of hydroxyapatite poly-L-lactide acid (“u-HA/PLLA”), placed into the trabecular bone of the sternum [21]. The device, mixed with the cortical bone, formed a truss structure and provided great stability. u-HA/PLLA is a bioabsorbable material with great biocompatibility and osteoconductivity, binding directly to the bone tissue and promoting osteosynthesis.

This material could be used in patients with metal allergies, and, as the trial showed, no adverse events have been found. Moreover, corrugated plates improved sternal healing, increasing sternal stability and suppressing bone displacement, in particular in high-risk patients. Fixsorb wave and wire cerclage should be used in fragile patients to reinforce the sternum, avoiding bone dehiscence. Furthermore, it is easy to apply and less expensive than RPF [22].

u-HA/PLLA might be used in several devices, such as screws or pins [22]. Sternal pins may reduce the antero-posterior displacement of the sternum, achieving an adequate approximation of the bone’s edges. The clinical benefit of the pins should be enhanced using u-HA/PLLA, leading to an earlier healing process, and reinforcing sternal closure without adverse infectious reactions.

### 4.2. Flexigrip

Takami et al. developed a thermoreactive nitinol (“Flexigrip”) to provide better chest stability than conventional wiring [23]. Flexigrip is a nitinol clip made of nickel and titanium with a memory effect, characterized by full malleability, as it changes its shape at <10 °C, allowing for its insertion among the two halves of the sternum, and once rewarmed, it restores its original and definitive size, allowing for bone approximation (Figure 3). Flexigrip, according to authors, should be a valid alternative to the wires or plates for sternal closure in fragile patients, as it is not appropriate to use the screw on an unhealthy or osteoporotic bone [23].

### 4.3. Custom-Made “Neo-sternum”

Technology developments in 3D printing might affect surgical practice. The possibility to design a custom-made titanium neo-sternum is appealing. In the literature, several cases showed that the use of 3D-printed titanium prostheses might represent an innovative alternative for sternal dehiscence (Figure 3). This technology should be also applied for the reconstruction of the anterior chest wall in patients with extensive tissue disruption after cancer, like chondrosarcoma, when a deep resection is needed, leading to chest instability. The prosthesis provides a rigid support of the chest with brilliant cosmetic results, improving postoperative respiratory complications. The prosthesis’ shape is based on the patient’s CT scan, and it requires approximately 6 weeks for its production. However, the costs and time needed for its use do not allow for its application in everyday surgical practice [24,25]. 

Wang and colleagues designed a modularized sternal prosthetic kit made of titanium standardized components of different sizes and types. Depending on the patients’ characteristics, the prosthesis was assembled, during surgery, using standard modules of different parts of the sternum, ribs, or clavicles. This technology does not require preoperative manufacture, and it is easy to apply to show good results without local infections or sternal instability after the procedure [26]. 

A new bioengineered tissue skin equivalent, called Apiligraf [27], has been tested on sternal and leg wounds as an innovative treatment for wound healing. Apiligraf is a bilayer living skin tissue made of fibroblasts, contained in a bovine collagen matrix, and keratinocytes as an epidermal layer with no evidence of rejection to human antigens. The benefits of using Apiligraf for wound healing are the following: (i) it promotes angiogenesis; (ii) it stimulates multiple growth factors, cytokines, and matrix proteins; (iii) it represents a physical barrier against infections; and (iv) it produces high levels of human beta defensin-2, a group of proteins with antimicrobial properties, providing a biological barrier from infections. Moreover, the surgical technique of its application is quite simple to perform, reducing the in-hospital stay of patients and, consequently, hospital costs. 

### 4.4. Alternatives to Bone Wax

Different studies identified postoperative bleeding as a main factor of infection [28]. Pradeep and his colleagues [28] highlighted that alternative materials should be used for hemostasis, instead of bone wax, as they are followed by greater infection rates and impaired sternal healing. 

The bone wax is composed of bees’ wax and paraffin, and it is mostly used over the sternal edges thanks to its malleability. It prevents bleeding by occluding the Haversian canals in the cortical bone and the medullary spaces within the cancellous bone. Moreover, since bone wax is not adsorbed by spongiosa, it creates a mechanical barrier that may delay the healing process and may promote bacterial infections [5].

Several materials, such as Ostene [29,30,31,32] or BoneSeal [33], represent a valid alternative for bone wax because of their similar characteristics to hemostatic agents. 

Ostene is a biocompatible bone wax, made of alkylene oxide copolymer. Unlike traditional bone wax, Ostene is absorbable within 28–52 h, guaranteeing optimal sternum healing.

In a comparative study, female pigs undergoing midline sternotomy were treated with either bone wax or Ostene. The results showed that those treated with Ostene experienced better outcomes, in terms of lower rates of wound infections, especially when enriched with antibiotics, such as the use of gentamicin. 

BoneSeal is a bone wax made up of hydroxyapatite and poly (lactic acid). A pilot study compared the use of BoneSeal and bone wax (control group) highlighting the osteosynthesis properties of this material and showing a significantly higher rate of new bone formation in pigs treated with BoneSeal than the control group [33]. 

Hydroxyapatite, chitosan, and oxidized starch have been developed as alternative materials to bone wax thanks to their viscoelastic properties and biocompatibility. 

A study conducted in a rat model showed the biological properties of a chitosan scaffold containing periostin as a valid alternative to bone wax. Periostin, an extracellular matrix protein, has a pivotal role in bone formation, increasing the activity of osteoblast cells and reducing the levels of proinflammatory cytokines, like TNF-alfa and IL-6. These cytokines directly induce osteoclast activations, managing bone absorption and leading to bone disorders, such as osteoporosis. Moreover, in the chitosan–periostin group, they highlighted a greater level of OPG, a receptor released by osteoblast cells, and osteocalcin, a marker of osteosynthesis, compared with the control groups. These data showed how periostin might interfere with the osteoblast/osteoclast balance, enhancing the osteogenic process. This chitosan scaffold, also, has antibacterial properties, especially against Gram-positive bacteria, while the use of bone wax accelerates bacterial proliferation [34].

The pregelatinized starch has been used to enhance the stiffness and smoothness of hemostatic agents, like the Poly (ethylene glycol) (PEG)-poly (propylene glycol) (PPG)- PEG copolymer. PEG combined with pregelatinized starch creates a physical barrier, making it easier to handle than traditional bone wax. It is a resorbable bone wax that does not affect the sternum healing process, as an in vivo analysis showed. PEG should be fixed with several elements taking advantage of their own characteristics, such as PEG–calcium phosphate which presents hydrophilicity, malleability, and cohesiveness better than bone wax. In addition, an antibacterial drug vancomycin should be incorporated into PEG, guaranteeing an antibacterial effect [28]. 

Gels could be used as a hemostatic agent for their injectable characteristics. An in vivo study showed its effects on developing a gel, based on chitin–fibrin, combined with tigecycline nanoparticles. The gel, applied on the sternal halves, releases the antibiotic for 3 weeks, reducing postoperative infections [28]. However, future research is warranted to confirm antibacterial effects.

## 5. Bone Adhesives

Bone adhesives for median sternotomy surgery need to have a number of properties in order to be considered ideal. Firstly, they need to be easy to apply, either in the form of a ready-to-use adhesive or one that requires minimum preparation at the point of care. Secondly, they need to set or cure quickly, ideally matching the time required for sternum closure. Thirdly, they should have a high adhesion strength to bone and sternal prosthetic materials. Fourthly, they should be flexible after curing to allow for some movement of the sternal halves during the healing process, dissipating the mechanical forces that would otherwise act on the adhesive bond. Fifthly, they should be biocompatible and not release any toxic components that could come into contact with the mediastinal tissues or the circulating blood. Finally, they should assist in the healing of sternal fractures, either by promoting the formation of callus at the fracture site or by themselves acting as fracture fillers [29,30,31,32].

Medical acrylate-based adhesives are widely used for bonding fragments for medical reasons and are suitable for polymeric additives to soften the adhesive composition. A problem of the modifications of medical acrylate-based rigid adhesives using polymer-based additives as fillers to achieve the flexibility of the composition and to improve the biomechanical and handling characteristics of these adhesives is stated. There are commercially available flexible composite materials; however, they are not suitable for the preparation of adhesives that could provide the strength of bonding for the bone fragments connected with the pressure during the sternum closure [33,34].

To increase flexibility and prevent a decrease in cohesion strength, polymer-based composites containing a solid cross-linked polymeric microstructure within a liquid monomeric continuous phase were designed as bone adhesives. Bend testing of the prototype adhesives during setting demonstrated a transition from high flexibility (modulus of ≤29 MPa) to low flexibility (modulus of ≥544 MPa) upon partial conversion of the liquid phase into the solid microstructure. Subsequently, lap shear testing with the well-designed double-curing bone adhesive optimized for penetration into the cancellous bone of the sternum showed significantly enhanced bonding strength. The flexural testing of this bone adhesive after complete curing indicated sufficient flexibility [35,36,37]. 

To investigate the mechanical properties of bone adhesives for cardiac surgery after sternotomy, two major concerns were considered in the design and testing of the adhesives after mixing and during setting. The first concern was adhesive brittleness. Although brittle adhesives rapidly form a rigid bond, the resulting bond does not deform much before breaking. As the patient’s thorax deforms about 2% during breathing, and considerable additional deformation occurs during daily activities, the adhesive must be sufficiently flexible to accommodate such movement to avoid rigid failure of the bond. The second concern was adhesive weakness. If an adhesive is very strong but the bond to the bone is weaker, the bond will break before the adhesive does. Therefore, both the adhesive-/bone-bonding strength and the adhesive cohesion strength should be high. However, solid adhesives often do not penetrate into the porous structure of bone, resulting in a weak adhesive/bone bond, and adhesive porosity usually decreases the bond strength [35,36,37,38,39].

## 6. Fibrous Sheets and Electrospun Fibers

In preclinical models, the hemostatic and osteosynthesis effects of cotton-like hydrophilic hydroxyapatite (HA) sheets have been shown. After 4 weeks of its application, radiographic and pathologic analysis showed a good healing process without the presence of collagen fibers compared with the control groups. The advantage of using it is its ready-to-use state, without increasing surgical time [40]. 

An electrospun scaffold made of poly (lactic acid) (PLLA) fibers mixed with hydroxyapatite nanoparticles was tested in a rabbit model showing a reduction in postoperative complications and a greater rate of sternal healing. The electrospun scaffold has biological and mechanical properties similar to native collagen, guaranteeing cell growth and differentiation into the bone lineage. PLLA, instead, thanks to its biocompatibility, might be combined with inorganic elements, like calcium, improving its biological properties as osteoconductive materials. This material represents a biological signaling, allowing for the differentiation of stem cells into osteoblasts, when interposed at the fracture line. It might mitigate the inflammatory reaction of the healing process, leading to tissue regeneration, as a study conducted by Rainer et al. [41] reported, showing the presence of a higher number of differentiated bone cells and new bone trabeculae in the treated group than the control one.

## 7. Growth Factor Therapies

Several studies underlined how growth factors might have an important role in the bone healing process, stimulating cell proliferation and differentiation. However, these growth factors have a short biological half-life, so a drug-delivery system (DDS) is required. The use of a gelatin hydrogel as a DDS allows for a controlled release of growth factors, enhancing their absorption and biological activity. Moreover, gelatin is converted into amino acids after its application, without adverse reactions [40,42]. 

PRP (platelet-rich plasma) gel is an excellent alternative for bone wax thanks to its osteogenic effect, promoting bone healing by acting at different stages of the process. Many studies conducted on sternal halves of diabetic patients highlighted a greater decrease in infection in patients treated with PRP gel than control group [43,44,45]. 

A meta-analysis by Kirmani et al. [46] analyzed the effect of the platelets gel. It guarantees a slow release of growth factors, an increase in mesenchymal stem cells, and a safe sternal healing process. Furthermore, when PRP is combined with fibrin or thrombin, it provides a lower rate of infections without side effects. 

Shibata [40], analyzing the use of PRP as a promoting factor for bone healing in a rabbit ischemic sternal model, showed that in the PRP+gel group, the mesenchymal stem cells were higher than the other groups, leading to a faster healing process. In fact, the PRP induced a differentiation of stem cells into pre-osteoblasts and osteoblasts, playing a key role in osteosynthesis.

Gallo and his colleagues [47] evaluated the use of autologous plasma-rich growth factors (PRGFs) as an alternative for bone healing. In particular, they applied PRGFs over the sternum of sheep, who had undergone median sternotomy, and they analyzed its effects after a nine-week follow-up. New trabecular bone tissue was discovered on the sternum treated with PRGFs, instead, and in the control group, they found only cartilaginous areas. The activation of platelets releases adhesive protein and growth factors, such as transforming growth factor beta, basic fibroblast growth factor, and epidermal growth factor, leading to a faster and better healing process than the control group. PRP is a valid material for tissue healing but more studies in the human clinical setting are needed.

The basic fibroblast growth factor (bFGF) has angiogenic properties and, also, osteogenic ones. Several studies showed an accelerated sternal healing process when it was applied. However, when it was injected in free form, it did not guarantee a proper regeneration process because of its short-acting activity. Iwakura et al. discovered a “formula” that improves the osteogenic effects of bFGF when combined with a biodegradable hydrogel gelatin. In fact, the gelatin hydrogel sheet allowed for a controlled release of bFGF, enhancing sternal regeneration, and it has been absorbed by the body without any harmful effects. Iwakura et al. [42,48,49] analyzed the effects of bFGFs in Wistar rats when it was applied to the posterior part of the sternum. The group, treated with a bFGF–gelatin hydrogel sheet, underwent bilateral internal mammary artery harvesting, leading to a decrease in blood flow in the sternum. The control group, instead, did not receive the growth factor, and another group kept BIMA. The first group showed an increase in blood vessels more than the control groups, thanks to the angiogenesis effect of bFGFs. Their results showed how the bFGF–hydrogel gelatin remains in situ for more than 34 days after its application with therapeutic effects, enhancing bone regeneration by increasing the number of osteoblasts around the sternal perimeter [49]. A similar study was conducted by the same group in diabetic rats, highlighting the osteogenic effects of bFGF–hydrogel gelatin in diabetic rats that underwent BIMA harvesting. BIMA grafts have been avoided in diabetic patients because of the high risk of deep sternal infections. However, the use of bFGF enhances bone healing by increasing peristernal blood flow thanks to its angiogenic properties. The use of bFGF mixed with a rigid control of blood glucose levels plays a key role in bone regeneration, highlighting how it might be feasible to harvest bilateral internal mammary arteries also in diabetic patients, improving postoperative outcomes after CABG compared with IMA graft [48].

## 8. Biophysical Stimulation Techniques

Pioneering applications of human endometrial stem cells (hEnSCs) have been recently reported in myocardial stimulation, and this might also have potential implications in bone healing. hEnSCs were encapsulated in an alginate hydrogel and subjected to a unique and innovative bioreactor modality that applied shear stress to stimulate cardiac patch fabrication [49,50,51]. This biohybrid has demonstrated remarkable promise in improving the integration of the implanted cardiac patch in clinical applications, particularly for the critical task of repairing myocardial infarction. The use of bioreactor stimulation plays a crucial role in ameliorating the cardiac behavior of these patches. By subjecting the encapsulated hEnSCs to controlled shear stress, researchers have successfully enhanced the functionality and compatibility of the patches before their implantation in the infarcted area. This innovative approach has not only propelled the field of regenerative medicine forward but has also opened up new avenues for effective bone regeneration. It is worth noting that the hEnSCs used in this study undergo a remarkable process of permanent differentiation, adapting seamlessly to become cardiomyocyte-like cells soon after mechanical preprocessing. This transformative process highlights the immense plasticity and regenerative capacity of these stem cells, further emphasizing their potential in cardiac tissue engineering [49,50,51,52,53].

## 9. Cell-Based Therapies

Utilization of bone morphogenetic protein 7 significantly augmented the effectiveness of stromal cell-based therapy [54,55]. Additionally, numerous studies have implemented a diverse range of scaffolds and supplementary agents to enhance the efficacy of MSCs. Bioglass/carnation-derived biodegradable sternal scaffold was skillfully seeded with mesenchymal stem cells and bone morphogenetic protein 7 [54,55]. Remarkably, this sternal site engineering scaffold achieved exceptional bone regeneration of the thoracic cage encompassing the sternum, exhibiting remarkable stability and strength. Injectable bone substitutes are composed of a calcium sulfate/alginate matrix, which was cleverly co-cultured with adipose-derived mesenchymal stem cells and endothelial cells (EPCs). Fascinatingly, this innovative approach triumphed in the formation of pre-vascular networks, paving the way for remarkable advancements in the field [54,55]. 

Cell-based therapies are emerging as a remarkably promising and innovative method for sternal bone regeneration, presenting immense potential in the field of medical advancements. These therapies encompass a diverse range of applications, including the utilization of mesenchymal stem/stromal cells, endothelial cells, osteoblasts, adipose-derived stem cells, and platelet-rich plasma, all of which contribute to the overall success of the regenerative process [54,55]. Among these various approaches, mesenchymal stem/stromal cells, along with their differentiated forms, have garnered significant attention and exploration, particularly within the realm of tissue-engineered constructs. Extensive research has revealed that mesenchymal stem/stromal cells possess the ability to exert profound influences upon the immune responses within the sternal wound, thereby playing a pivotal role in the fight against infection. Importantly, these cells are derived from a multitude of tissues, such as the bone marrow, adipose tissue, and even the umbilical cord, fostering a vast array of possibilities for their application in regenerative medicine. A notable study conducted by the esteemed Donndorf group elucidated the potential of utilizing autologous bone marrow—or adipose tissue-derived stromal cells—successfully seeded within a biodegradable polymer construct. This innovative approach served to considerably augment the osteogenic, angiogenic, and wound healing capacities of the debrided sternal bone, representing a significant leap forward in the realm of regenerative therapies. In light of these findings, it becomes apparent that the utilization of cell-based therapies holds immense promise for the successful and efficient regeneration of sternal bone. The various cell types, including mesenchymal stem/stromal cells, endothelial cells, osteoblasts, adipose-derived stem cells, and platelet-rich plasma, collectively contribute to the multifaceted nature of these therapies, providing a comprehensive approach to address the complex challenges associated with sternal bone regeneration [54,55].

The pros and cons of the most promising future enhancers of sternal wound repair are summarized in Table 3. The absence of consensus in the literature and the lack of adequately powered randomized studies comparing sternal closure methods determines the inability to perform computation for previous studies without leaving significant heterogeneity and bias.

## 10. Conclusions

The management of sternal wound complications after cardiac surgery is a crucial aspect of guaranteeing a successful outcome for patients. Sternal wound healing is rather challenging due to the complicated stress environment, which is the reason why stainless steel wires are the common option in surgical practice. Considering the comparative performance of each technique of sternal repair, including efficacy, durability, and costs, no “one-size-fits-all” approach seems reasonable. Indeed, the closure technique should be inspired and tailored to each patient considering the lack of specific guidelines. The operative approach should consider the presence of risk factors for impaired healing, i.e., female sex, obesity, diabetes, severe chronic pulmonary disease, bilateral harvesting of the mammary artery, and off-line sternotomy. The single-wire technique seems to be the most useful method in routine patients, and in case of risk factors, rigid plate fixation seems to produce optimal results even in high-risk patients. However, the use of plates might be limited by center availability and funds. When unavailable, sternal plates can be replaced by more advanced techniques such as weaves. However, any stainless steel technique might be inappropriate in case of extremely diseased sternum, and various innovative techniques and materials have been developed to improve sternal closure and promote bone healing, especially because of the high mortality risk of mediastinitis in fragile patients. These new techniques and materials aim to improve sternal healing, reduce infections, and enhance patient outcomes. The current literature, considering both preclinical studies and pioneering clinical applications, is encouraging (Table 4). Further studies and clinical trials are needed to evaluate the long-term outcomes and efficacy of these methods in different patients. Tailored approaches to sternal closure and wound management based on patient-specific risk factors and characteristics may lead to better outcomes and quality of life for patients undergoing cardiac surgery [56].

## Figures and Tables

**Table 1:** Standard techniques for sternal reconstruction after cardiac surgery.

Wire Closure Style	Modeled Illustration	Advantages	Disadvantages
Alternating peri-sternal and trans-sternal	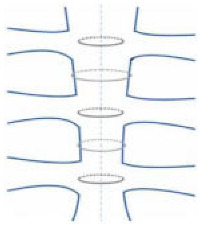	Superior strength and stability	Can easily injure osteoporotic bones
Single trans-sternal	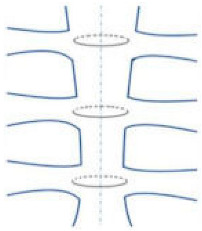	Easy to use Allows for a good body union in most patients	The twisted free ends of the wire may penetrate the sternum (due to osteoporosis or other factors) Requires seven or more wires
Single peri-sternal	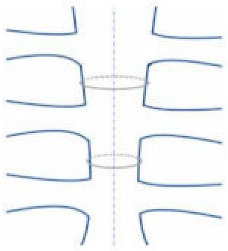	Reduces risk of deep sternal wound infection by reinforcing the sternum Safe for solid internal fixation Sternal stability was higher in single wire vs. figure-of-eight wire in high-risk obese patients	Requires seven or more wires
Figure-of-eight	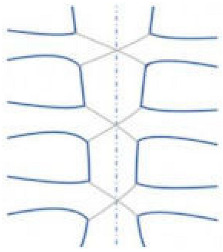	Potential benefit in osteoporotic bone Biomechanical benefit (larger surface area)	Conflicting results in the literature
Modified figure-of-eight	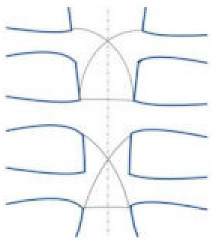	Effective and safe method for limiting sternal dehiscence by limiting the penetration in the intercostal spaces	Conflicting results in the literature
Longitudinal parasternal (Robicsek cage)	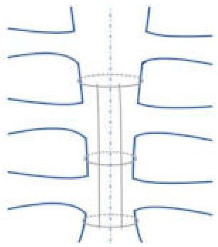	Used for high-risk patients (chronic pulmonary disease, obesity, bilateral mammary artery harvesting, diabetes, off-midline sternotomy, and patients undergoing reoperations)	Increased risk of sternal de-vascularization due to antero-posterior compression

Adapted from Al-Abbassi et al. [1].

**Table 2:** Preoperative, intraoperative, and postoperative risk factors for sternal wound complications.

Preoperative Factors	Intraoperative Factors	Postoperative Factors
Macromastia	Bilateral internal mammary artery harvesting	Reoperation
Large chest circumference	Paramedian sternotomy	Blood transfusion
Obesity	Sterility breaks	Longer hospital stay
Active smoking	Prolonged operation time	
Diabetes mellitus	Poor closure technique	
Osteoporosis		
Chronic pulmonary disease		
Corticosteroid use		

Adapted from Nenna et al. [5]

**Table 3:** Pros and cons of most promising future enhancers of sternal wound repair.

Concept	Materials	Positive Factors	Negative Factors	References
**Bone Adhesives**	Medical acrylate	Easy to apply Ready to use Biocompatible and does not release any toxic components	Solid adhesives often do not penetrate into the porous structure of bone Transition from high flexibility (modulus of ≤25 MPa) to low flexibility (modulus of ≥500 MPa)	[35,36,37,38,39]
**Fibrous Sheets and Electrospun Fibers**	Cotton-like hydrophilic hydroxyapatite (HA)	Ready to use Does not increase surgical time	**/**	[40]
	Electrospun scaffold poly (lactic acid) (PLLA)	Reduction in postoperative complications and a greater rate of sternal healing Properties similar to native collagen Mitigate the inflammatory reaction	**/**	[41]
**Growth Factor Therapies**	Gelatin hydrogel with drug-delivery system (DDS)	Release of growth factors Gelatin is converted into amino acids after its application, without adverse reactions	**/**	[42]
	PRP (platelet-rich plasma)	Excellent alternative for bone wax Promoting bone healing Combined with fibrin or thrombin, it provided a lower rate of infections without side effects Angiogenic properties	When injected in free from, it did not guarantee a proper regeneration process because of its short-acting activity	[43,44,45]
**Cell-Based Therapies**	Mesenchymal stem cells (MSCs)	Contribute to the overall success of the regenerative process	**/**	[54,55]

**Table 4:** Novel biomaterials for sternal wound closure: summary of preclinical and clinical application (adapted from Pradeep et al. [28]).

Biomaterial	Use	Action
Calcium sulfate with hydroxypropyl methylcellulose (HPMC) or sodium alginate	preclinical	The released calcium ions from the material can activate the coagulation cascade when it comes in contact with the blood thereby preventing bleeding.
Chitin–fibrin gel incorporated with tigecycline nanoparticles	preclinical	An adhesive gel with hemostatic properties and controlled drug release for 21 days.
Chitosan, oxidized starch, and hydroxyapatite	preclinical	Wax-like material with viscoelastic properties and biocompatibility.
Electrospun material Poly (l-lactide)/hydroxyapatite	preclinical	The scaffold was found to enhance sternal healing in the rabbit.
Gelatin hydrogel sheet with PRP/beta –FGF	preclinical	PRP/beta –FGF release was found to enhance sternal healing
Hydroxyapatite sheet with beta-tricalcium phosphate	preclinical	The sheet was sandwiched between the sternal halves, and their effects on sternal healing were studied in the canine model.
PEG- PPG- PEG with pregelatinized starch	preclinical	Has tamponade effects to prevent bleeding and shows good biocompatibility with osteoblast cells.
Poly-(ethylene glycol)–calcium phosphate cement with pregelatinized starch	preclinical	Like bone wax, it acts as a physical barrier to prevent bleeding. Tetracalcium phosphate provides osteogenic effects.
Polydopamine-co-acrylate and hydroxyapatite nanoparticles	preclinical	Material with controlled setting time, which can be used to enhance sternal healing.
Tricalcium silicate, 58S bioglass, chitosan, and carboxymethyl cellulose	preclinical	Injectable wax-like material with osteogenic and hemostatic effects.
AVITENE (microfibrillar collagen and antibiotic-containing fibrin glue)	clinical	It was applied at the sternum and was able to prevent bleeding and control infection.
BONESEAL (polylactic acid and hydroxyapatite)	clinical	It can act as a physical barrier against bleeding and enhance bone healing.
CALLOS (calcium phosphate cement)	clinical	It prevents bleeding and enhances better sternal and soft tissue healing with complete absorption of the material.
COLLOTAMP (gentamicin-impregnated collagen sponge)	clinical	The gentamicin-impregnated sponge placed in between the sternal halves helps in preventing infection.
HEMOBLAST (porcine collagen, bovine chondroitin sulfate, and human pooled plasma thrombin)	clinical	Explored for their effects in controlling sternal bleeding.
KRYPTONITE (castor oil-based adhesive)	clinical	It can enhance sternal union and stability.
OSTENE (alkylene oxide copolymer)	clinical	Water-soluble bone wax acts as a physical barrier against bleeding and is completely resorbable.
SPONGOSTAN (gelatin powder with rifamycin powder)	clinical	It is applied on the bone and helps in controlling bleeding.
VIVOSTAT (fibrin sealant with batroxobin)	clinical	The hemostasis effect of the material was studied, and clotting was observed within 43 s.

## Data Availability

Not available (review article, no new data created).
